# Aging of the placenta

**DOI:** 10.18632/aging.204175

**Published:** 2022-07-13

**Authors:** Hongbo Qi, Liling Xiong, Chao Tong

**Affiliations:** 1 Department of Obstetrics, Women and Children’s Hospital of Chongqing Medical University, Chongqing 401147, China; 2 Department of Obstetrics, Chongqing Women and Children's Health Center, Chongqing 401147, China; 3 Department of Gynaecology and Obstetrics, Chengdu Women’s and Children’s Central Hospital, School of Medicine, University of Electronic Science and Technology of China, Chengdu 611731, China; 4 State Key Laboratory of Maternal and Fetal Medicine of Chongqing Municipality, The First Affiliated Hospital of Chongqing Medical University, Chongqing 400016, China

**Keywords:** aging, placenta, advanced maternal age

The placenta is a temporary organ that only exists during pregnancy. It is tasked with facilitating the exchange of oxygen, nutrients, and metabolic waste between mother and fetus. This organ also secretes many essential pregnancy-related hormones, growth factors, and cytokines that support fetal development. Premature deterioration or dysfunction of the placenta leads to pregnancy complications, compromised fetal development, or even failure of pregnancy.

The development of the placenta starts from the blastocyst, which adheres to the endometrial epithelium in a process called implantation. The outer layer of the pre-implantation embryo, the trophectoderm (TE), must first differentiate into cytotrophoblasts (CTBs), which then transforms into syncytiotrophoblasts (STBs) and extravillous trophoblasts (EVTs) through syncytialization and differentiation. These vital trophoblasts comprise the placenta, along with a considerable number of fibroblasts, decidua cells, immune cells and vascular cells [1].

As gestation progresses, the placenta undergoes senescence. Generally, this process is necessary to detach the placenta from the uterine wall following parturition, eventually allowing blood vessels to close (to prevent hemorrhage) and the uterus to regain pre-pregnancy size and shape. In recent years, our knowledge of aging of the placenta has largely expanded. A better understanding of this physiological process will lead us to develop successful interventional strategies to combat premature senescence of placenta and related diseases.

Senescence of placenta is also marked by gradual elevations in the expression of p53, p21 and p16. However, cellular senescence can have mixed effects on the placenta. On one hand, cell-fusion-induced senescence of syncytiotrophoblasts is essential for the development of the placenta and maintaining its function [2]. On the other hand, premature senescence of EVTs is associated with placental maldevelopment, which leads to blood hypoperfusion and consequently unfavorable perinatal outcomes such as preeclampsia, fetal growth restriction, preterm birth, and stillbirth. Importantly, the progress of placental senescence varies among individuals. Recent studies suggest that the placenta of advanced maternal age (AMA) has the pathological characteristics of premature senescence from an early stage of pregnancy, which is associated with higher rates of delayed villous maturation, fetal vascular malperfusion and maternal vascular lesions [3].

Senescence may be induced prematurely in the placenta by various factors, including oxidative stress (OS), telomere attrition, DNA damage, and mitochondria and protein dysfunction [4]. Conversely, the senescence-associated secretory phenotype (SASP) of senescent placental cells induces microenvironmental inflammation, which may further reinforce telomere shortening, oxidative stress, DNA damage, and mitochondrial and protein dysfunction. Nevertheless, the contribution and underlying mechanisms of cell-cross talk to aging of the placenta remains largely unknown.

A recent study by Xiong and colleagues demonstrated silent information regulator 1 (SIRT1) deficiency results in premature senescence of trophoblasts and increased acetylation of vimentin, thereby impairing the invasion and migration of trophoblasts by suppressing epithelial-mesenchymal transition (EMT) [5]. Chen et al. reported that AMA is associated with the loss of another antiaging molecule α-klotho in trophoblasts, leading to a senescent phenotype and compromised invasiveness by repressing the transcription of cell adhesion molecule (CAM) genes [3]. Nevertheless, the senescence of other types of placental cells and their involvement in how the placenta ages have yet to be identified. Furthermore, emerging evidence has revealed that the early events of pregnancy, including embryo attachment, decidualization, uterine luminal epithelium-stroma communications, invasion, etc. may profoundly modulate placental aging; further validations are warranted.

Noticeably, AMA pregnancy is rapidly increasing worldwide, which results in significantly higher incidences of pregnancy complications and cesarean delivery compared to pregnancy outcomes observed in younger women [6]. The increase in AMA pregnancy has led to research and development of anti-aging approaches for the placenta, though these approaches are still novel and have yet to be validated. One promising approach involves therapeutic strategies of scavenging reactive oxygen species (ROS), such as N-acetyl-L-cysteine and transplantation of mesenchymal stem cells (MSCs). These strategies effectively reduce telomere shortening, telomere fusion and chromosomal instability in the female reproductive system and improve female fertility as well as early embryo development [7]. These findings shed light on the management of OS in placental rejuvenation.

Additionally, resveratrol has been found to improve telomerase activity along with telomere length and longevity of the ovarian follicular reserve through the enhanced expression of SIRT1. The SIRT1-specific activator, SRT1720, remarkably increases the migration and invasion ability of trophoblast cells *in vitro*. These results indicate that sirtuin-activating compounds (STACs) show promising in alleviating AMA-associated senescence in the placenta.

Clearance of senescent cells is believed to be beneficial for resisting aging. Particularly, senolytics, a class of drugs that selectively stimulate apoptosis in senescent cells, effectively postponed the aging phenotype in animal experiments and could be useful to treat aging in the placenta. Among them, quercetin, a plant-derived bioflavonoid that possesses antioxidative and anti-inflammatory properties, could reduce the age-associated accumulation of ROS and decline of sirtuin expression [8]. Another potential candidate drug, Dasatinib, a small molecule inhibitor of multiple kinases including SRC-family protein tyrosine kinase (SFK) and discoidin domain receptors (DDR2), preferentially slowed the growth and induced apoptosis of senescent cells. However, eliminating senescent cells introduces certain risks for error in critical physiological events of gestation, such as decidualization and embryo implantation. These phases rely on senescent cells derived from the local proinflammatory microenvironment. As a result, different senotherapies to treat or prevent placental aging should be carefully tailored according to desired effects.
